# Extracellular Matrix-Derived Matrikines: Circulating Peptides as Candidate Mediators of Lung-to-Brain Signaling

**DOI:** 10.3390/ijms27073339

**Published:** 2026-04-07

**Authors:** Andis Klegeris

**Affiliations:** Laboratory of Cellular and Molecular Pharmacology, Department of Biology, University of British Columbia Okanagan Campus, Kelowna, BC V1V 1V7, Canada; andis.klegeris@ubc.ca

**Keywords:** elastin-derived peptides, elastokines, endorepellin, Gly-His-Lys (GHK), Ile-Lys-Val-Ala-Val (IKVAV), lung–brain axis, matrikines, Pro-Gly-Pro (PGP), Tyr-Ile-Gly-Ser-Arg (YIGSR), Val-Gly-Val-Ala-Pro-Gly (VGVAPG)

## Abstract

Recent studies support the concept of a bidirectional lung–brain axis. While neural, immune, and microbial pathways are increasingly recognized in lung-to-brain communication, the role of matrikines—bioactive peptides generated by extracellular matrix (ECM) proteolysis during remodeling—in this inter-organ communication remains underexplored. This review highlights matrikines originating from the lung, particularly the collagen-derived tripeptide Pro-Gly-Pro (PGP) and the elastin-derived hexapeptide Val-Gly-Val-Ala-Pro-Gly (VGVAPG), as potential mediators linking pulmonary pathology with neurological outcomes. The lung is rich in ECM proteins, and inflammatory conditions such as chronic obstructive pulmonary disease (COPD) and emphysema trigger proteolytic activity by matrix metalloproteinases (MMPs) and neutrophil elastase, releasing matrikines into circulation. Under conditions of blood–brain barrier (BBB) dysfunction, they may access the central nervous system (CNS), where they influence neurons, microglia, and astrocytes, modulating neuroinflammation, autophagy, and synaptic integrity. While PGP can exhibit context-dependent neuroprotective effects, its acetylated form and VGVAPG are associated with neurotoxicity, Tau hyperphosphorylation, and microglial activation. Additional matrikines, including Gly-His-Lys (GHK) and endorepellin, may further modulate CNS homeostasis. Collectively, these findings support lung-derived matrikines as circulating mediators of lung-to-brain signaling, providing a novel mechanistic framework linking chronic pulmonary inflammation to neuropathologies, such as stroke and neurodegenerative disorders, and highlighting potential targets for therapeutic intervention.

## 1. Introduction

The link between the gut and mental states has been noted for over a century, but rigorous experimental investigation of gut-to-brain signaling only emerged in neuroscience in the late 20th and early 21st centuries. Research accelerated after landmark germ-free animal studies in the early 2000s demonstrated that gut microbiota can influence stress responses and hypothalamic–pituitary–adrenal (HPA) axis activity, with the vagus nerve playing a key role in this communication (reviewed in [1,2,3]). In contrast, systematic exploration of lung-to-brain signaling is only beginning. Recent studies and reviews describe bidirectional communication between the respiratory system and the central nervous system (CNS) through neural pathways, such as the vagal and glossopharyngeal nerves, as well as through hypoxemia-linked mechanisms—including hypoxia-induced inflammatory signaling and cerebrovascular dysregulation—that may influence brain function in both health and disease (reviewed in [4,5,6]). Together, these findings highlight the still nascent conceptualization of a lung–brain axis. The potential pathophysiological significance of lung-to-brain signaling is underscored by associations between pulmonary pathologies, including acute respiratory distress syndrome (ARDS), pneumonia, asthma, and chronic obstructive pulmonary disease (COPD), and neurological outcomes. These include cognitive impairment, stroke sequelae, and neurodegenerative disorders, notably Alzheimer’s (AD) and Parkinson’s diseases (reviewed in [5,6,7,8]). Although causal links between lung disease and neurological disorders remain challenging to demonstrate in humans, several recent lines of evidence support a directional influence. Genetic causal inference studies suggest that airway disease phenotypes may influence brain structural networks, consistent with a lung-to-brain axis [9]. Experimental work shows that cigarette smoke-induced COPD leads to increased blood–brain barrier (BBB) permeability, neuroinflammation, and cognitive deficits in animal models [7]. Large cohort studies also link poor lung function with accelerated cognitive decline and markers of brain aging [10]. These observations imply temporal and mechanistic relationships beyond cross-sectional associations, even if definitive causal proof in humans remains limited.

Beyond neural pathways, immune and inflammatory signaling and microbe-associated mechanisms are increasingly recognized as important contributors to both gut- and lung-to-brain communication. These processes imply diffusible signaling between peripheral organs and the CNS, with several distinct classes of mediators identified. In lung-to-brain signaling, molecules released by lung epithelial cells, resident immune cells, and, more recently, the lung microbiota-associated molecular signals are considered prominent contributors. Recent advances in single-cell RNA sequencing (scRNA-seq) have demonstrated that the composition and functional states of cells comprising the human airway epithelium vary along the proximal–distal axis and are influenced by the presence of disease [11]. Lung epithelial cells contribute to local and systemic signaling by releasing cytokines, chemokines, and growth factors, which may also serve as mediators in lung-to-brain communication. Under inflammatory conditions, airway epithelial cells secrete a range of immune signaling molecules capable of modulating the activity of key CNS cell types, including interleukin (IL)-1β, C-X-C motif chemokine ligand 8 (CXCL8/IL-8), C-C motif chemokine ligand 2 (CCL2/MCP-1), tumor necrosis factor (TNF), and transforming growth factor (TGF)-β [12,13]. Lung epithelial cells also release nanosized extracellular vesicles (EVs) under both physiological and pathological conditions. The cargo of these EVs, comprising bioactive molecules such as proteins and RNAs, reflects the functional state of the cells from which they originate. Because EVs can be detected in both bronchoalveolar lavage fluid and blood, they have been proposed as a potential means of communication between the lung and the brain, with the capacity to influence CNS functions [14]. Beyond the epithelium, healthy lungs harbor a diverse population of immune cells, including alveolar macrophages, dendritic cells, neutrophils, mast cells, innate lymphoid cells, and resident T and B lymphocytes, that produce basal levels of cytokines and chemokines. This repertoire of immune mediators can change dramatically in pulmonary pathologies. Such immune mediator-driven signaling likely represents one of the key mechanisms underlying lung-to-brain communication [5,6,13].

Although the gut has long been recognized as densely colonized by diverse microbial communities essential for host physiology, the lungs were historically considered relatively sterile in comparison. Only in recent years has it become established that healthy lungs harbor distinct, low-biomass microbial communities, which are dynamically altered in disease states such as asthma, COPD, cystic fibrosis, and lung cancer (reviewed in [15,16]). Emerging evidence indicates that lung microbes can produce bioactive metabolites, including tryptophan derivatives, short-chain fatty acids (SCFAs), such as acetate, propionate, and butyrate, as well as other microbially derived compounds, including adenosine and polyamines. These metabolites are known to shape peripheral immune responses and may also contribute to lung-to-brain communication through systemic immune signals and neuroimmune mechanisms, including cytokine signaling and modulation of CNS immune cells (reviewed in [16,17,18]).

Another important consideration when evaluating signaling between the lung or other peripheral organs and the CNS is the integrity of the BBB, which under physiological conditions is highly restrictive to circulating macromolecules and plays a central role in maintaining brain homeostasis. It is well-known that systemic inflammatory responses, characterized by elevated circulating cytokines (e.g., TNF, IL-6, IL-1β), can activate endothelial cells at the BBB and lead to degradation of tight junction proteins such as claudin-5 and occludin, thereby compromising barrier integrity. Experimental evidence shows that peripheral immune signaling can disrupt the BBB, allowing microbial products, inflammatory cytokines, and other systemic mediators to enter the CNS, where they contribute to neuroinflammation and altered neural homeostasis (reviewed in [18,19,20,21]). Similarly, growing evidence indicates that certain lung disorders are associated with increased BBB permeability (reviewed in [5]). For example, in a murine experimental model of COPD, lung injury caused by intratracheal administration of porcine pancreatic elastase is accompanied by BBB hyperpermeability and disruption of endothelial tight junctions, potentially facilitating the entry of circulating inflammatory mediators into the CNS and contributing to neurovascular dysfunction and cognitive symptoms [8,22]. Furthermore, clinical imaging studies in patients with advanced lung cancer show increased BBB leakage in multiple brain regions compared with healthy controls, even in the absence of visible brain metastases, and this increased permeability correlates with cortical thinning and reduced volumes of subcortical structures [23]. Moreover, acute lung infection with the bacterial pathogen *Pseudomonas aeruginosa* in an experimental murine model induces neuroinflammation and BBB dysfunction, characterized by increased paracellular permeability and reductions in key tight junction proteins (e.g., vascular endothelial cadherin and claudin-5). These changes are accompanied by behavioral alterations and are thought to be mediated, at least in part, by systemic cytokines [24].

It is also important to note that increased BBB permeability is a well-established feature of many neurological diseases and brain pathologies. Its disruption has been documented in conditions such as AD, Parkinson’s disease, multiple sclerosis, stroke, epilepsy, neurotrauma, and viral encephalitis, where it contributes to neuroinflammation, immune cell infiltration, and neuronal dysfunction by allowing normally excluded blood-derived molecules and cells to enter the CNS (reviewed in [25,26]). Increased permeability of this barrier, whether caused by primary brain pathology or by excess inflammatory stimuli from systemic inflammation or other peripheral disease, can facilitate signaling between peripheral organs, including the lungs, and the CNS (reviewed in [5,6]). Under conditions of compromised BBB, all of the circulating mediators discussed above, including EVs, cytokines, chemokines, and microbial metabolites may traverse the BBB more readily, thereby enhancing communication between peripheral sites of pathology and the brain. Importantly, these mediators likely represent only a subset of the molecular signals involved in such lung-to-brain communication, and the known repertoire will almost certainly expand as new experimental evidence becomes available [5].

**Figure 1:**
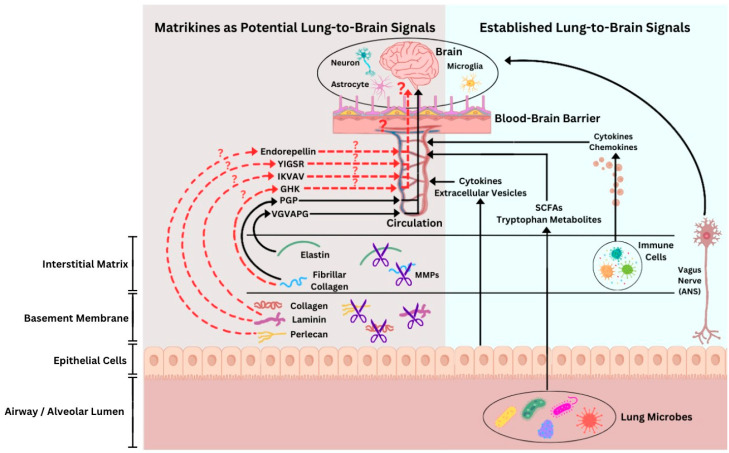
Matrikines as potential signaling molecules in lung-to-brain communication. Current evidence supports the collagen-derived Pro-Gly-Pro (PGP) and the elastin-derived Val-Gly-Val-Ala-Pro-Gly (VGVAPG) as candidate mediators of lung-to-brain signaling. Both these matrikines are generated in inflamed pulmonary tissue, can enter the systemic circulation, and may access the CNS under conditions of BBB disruption, where they can influence neuronal and glial cell function. Additional matrikines with reported actions on brain cells are depicted, although their lung origin and ability to reach the CNS are not yet defined. Established lung-to-brain communication pathways are illustrated for context, including the autonomic nervous system and mediators released by lung epithelial and immune cells, as well as lung microbes. Figure was created with Canva.com, https://canva.link/li91qw8s6kat4q7 (accessed on 3 April 2026). ANS, autonomic nervous system; MMPs, matrix metalloproteinases; SCFAs, short-chain fatty acids.

One additional class of signaling molecules that remains comparatively underexplored and warrants consideration in this context is matrikines. They are bioactive protein fragments generated by proteolytic cleavage of extracellular matrix (ECM) proteins, including collagens, laminins, perlecan, fibronectin, and elastin, and are increasingly recognized as regulators of inflammation, tissue repair, and intercellular signaling. Notably, the lungs, alongside organs such as the skin and vascular tissues, are sites where ECM remodeling is particularly prominent. Additionally, pathological contexts, including tumors, also display extensive ECM remodeling, which has allowed the diversity and biological functions of matrikines to be most thoroughly studied in these settings (reviewed in [27,28,29]). Within these environments, EVs may represent a potential mechanism for the systemic distribution of matrikines. While direct evidence that matrikines are packaged into EVs is currently lacking, matrikines themselves have been shown to induce EV shedding; for example, elastin-derived peptides (EDPs) promote tumor cell blebbing and EV release [30]. Given that EVs are established carriers of diverse bioactive cargo, including peptides and proteins, this raises the possibility that matrikines could be transported within EVs under specific physiological or pathological conditions (reviewed in [31]). This article highlights emerging evidence implicating select lung-derived matrikines as novel mediators of the lung–brain signaling axis (Figure 1).

## 2. Lung Extracellular Matrix and Matrikines

The concept of matrikines arose from early studies of ECM turnover in arterial and dermal connective tissues, where proteolytic degradation of elastin and collagen was shown to generate bioactive fragments with cellular signaling properties [28,29,32]. The lungs soon became a key organ of matrikine research, owing to its exceptionally high content of elastin and collagen, its exposure to intense proteolytic activity mediated by neutrophil elastase and matrix metalloproteinases (MMPs), and its susceptibility to chronic inflammatory conditions such as COPD, emphysema, and pulmonary fibrosis (reviewed in [33,34]). The lung ECM is a complex and dynamic network of structural and regulatory macromolecules, providing mechanical support for respiratory tissues as well as biochemical signals that regulate cell behavior, tissue homeostasis, and repair. It is organized into two principal compartments with distinct composition and functions. Basement membranes are thin, dense sheets of specialized ECM that underlie the airway and alveolar epithelium, composed predominantly of the non-fibrillar, network-forming collagen IV, laminins (the most abundant non-collagenous components), heparan sulfate proteoglycans, such as perlecan, and associated glycoproteins. In contrast, the interstitial matrix forms a three-dimensional network surrounding stromal cells and is particularly rich in fibrillar collagens (primarily type I and III) and elastin. These structural networks provide tissue strength, elasticity, and compliance essential for normal respiratory biomechanics, while also acting as a reservoir for growth factors and cytokines that shape local signaling and repair processes [32]. In this context, several lung-derived matrikines, most notably the collagen-derived peptide Pro-Gly-Pro (PGP) and the elastin-derived Val-Gly-Val-Ala-Pro-Gly (VGVAPG), have been shown to exert potent biological effects, including regulation of chemotaxis of neutrophils and monocytes, amplification of inflammatory signaling, and promotion of sustained tissue injury (reviewed in [33,34]). Notably, a growing body of evidence suggests that these same matrikines can influence specific functions of brain-resident cells (see Section 4 and Section 5), providing a conceptual foundation for their potential involvement in lung-to-brain signaling. It is important to note that collagen and elastin, from which PGP and VGVAPG are derived, are present at comparatively low levels in the healthy brain ECM relative to the lung (reviewed in [35,36]). Their abundance in CNS tissues increases primarily under pathological conditions, reinforcing the focus of this review on the role of PGP and VGVAPG in unidirectional lung-to-brain signaling. In addition to PGP and VGVAPG, several other established matrikines demonstrate biological activity affecting distinct CNS cell populations. These include the collagen-derived tripeptide GHK (Gly-His-Lys); laminin-derived peptides (LDPs) including YIGSR (Tyr-Ile-Gly-Ser-Arg) and IKVAV (Ile-Lys-Val-Ala-Val); and endorepellin, a C-terminal fragment of domain V of the heparan sulfate proteoglycan perlecan. Although definitive evidence is currently lacking, it is plausible that these four additional matrikines could be generated in the lung under physiological or pathological conditions and, following entry into the circulation and passage across a compromised BBB, modulate CNS cellular functions (Figure 1).

## 3. Brain Cell Types Affected by Matrikines

Signals arising from peripheral tissues such as the lung and gut can influence the brain not only through classical neural and humoral pathways, but also by engaging cellular signaling networks within the CNS. Once peripheral mediators, including cytokines, chemokines, microbial metabolites, EVs, and matrikines, access the cerebrospinal fluid (CSF) and brain parenchyma, they encounter a heterogeneous population of CNS cells whose coordinated responses determine local neuroimmune status, brain function, and overall tissue homeostasis (reviewed in [6,20,37]). All major CNS cell types—including neurons, microglia, astrocytes, and oligodendrocytes—are responsive to both peripheral and CNS-derived endogenous cues, although the downstream effects are cell type-specific.

Neurons, the principal information-processing cells of the CNS, integrate synaptic inputs and orchestrate cognitive and motor outputs. Neuronal homeostasis is highly sensitive to extracellular cues, including neurotransmitters, gliotransmitters, metabolic stress signals, inflammatory mediators, and ECM remodeling fragments. Dysregulated signaling in neurons contributes to synaptic dysfunction, altered electrophysiological properties, and degenerative cascades implicated in conditions such as AD and Parkinson’s diseases (reviewed in [19,35,38]).

Microglia, the resident innate immune cells of the brain, act as sentinels of the CNS environment. They continuously survey their surroundings and respond to pathogens, cellular debris, and damage-associated molecular patterns (DAMPs) by undergoing context-dependent activation and functional reprogramming. Reactive microglia release pro- and anti-inflammatory cytokines, phagocytose synaptic elements, and can modulate neuronal survival or degeneration depending on the local microenvironment. A variety of peripheral signals that reach the CNS can influence microglial activation states, migration, and effector functions, thereby shaping neuroinflammatory trajectories (reviewed in [20,37,39]).

Astrocytes, the most abundant glial population, support neurons metabolically, maintain ion and neurotransmitter balance, and contribute to the structural and functional integrity of the BBB. Astrocytes also produce cytokines and chemokines in response to injury or pathogens, participate in scar formation after tissue damage, and regulate synaptic remodeling. Microbiota-derived molecules and systemic inflammatory cues can modify astrocytic gene expression, redox balance, and interactions with neurons and immune cells (reviewed in [20,40,41]).

Oligodendrocytes are the myelinating cells of the CNS, responsible for insulating axons and supporting rapid electrical signal conduction. Consistent with neurons, microglia, and astrocytes, oligodendrocytes are responsive to inflammatory mediators, DAMPs, and bioactive peptides; these signals influence oligodendrocyte maturation, myelination capacity, and survival. Such responsiveness places oligodendrocytes within the broader network of CNS cells that translate peripheral cues into functional changes in brain circuitry. Dysregulation of oligodendrocyte function has been implicated in neuroinflammatory and neurodegenerative conditions, suggesting that circulating signals from peripheral organs, including the lung, could indirectly affect neuronal connectivity and CNS network integrity (reviewed in [42,43]).

Together, these four major CNS cell types—neurons, microglia, astrocytes, and oligodendrocytes—represent key targets for peripheral mediators crossing a compromised BBB and engaging local signaling networks. Matrikines released from peripheral organs during ECM remodeling, including those generated in the lung, have the potential to interact with receptors and signaling pathways in these cells. In doing so, they may contribute to neuroimmune activation, synaptic and structural remodeling, and altered neural function. Among the ECM-derived peptides discussed above, PGP and VGVAPG represent the most promising candidates for mediating lung-to-brain signaling, with accumulating evidence suggesting their capacity to influence the activity of neurons, microglia, and astrocytes.

## 4. PGP as a Candidate Lung-to-Brain Matrikine

### 4.1. Overview of PGP

The tripeptide PGP is generated by enzymatic degradation of collagen. It can undergo N-terminal acetylation via a non-enzymatic chemical reaction, forming N-acetyl-PGP (Ac-PGP), which often exhibits enhanced biological activity compared to unmodified PGP [44]. The PGP sequence is abundant in fibrillar collagen types I, III, and V, which are all major components of the lung extracellular matrix, but remains biologically inert while embedded within the triple-helical collagen structure. Liberation of PGP requires sequential proteolysis: MMP-8 and MMP-9 first cleave collagen into intermediate peptides, which are then further processed by prolyl endopeptidase to release free PGP. Structurally, PGP shares homology with chemokines CXCL1, CXCL2, and CXCL8, which are known to interact with the chemokine receptors CXCR1 and CXCR2 (reviewed in [34,44,45,46]).

### 4.2. Evidence of PGP Production in the Lungs

PGP and Ac-PGP have been directly detected in both human and murine lung tissue, demonstrating that the lung is a significant site of their generation. Levels of both matrikines are elevated in chronic inflammatory lung diseases, including COPD and cystic fibrosis, as well as following acute lung injury [34,45,47]. Relevant to lung pathophysiology, PGP generation under inflammatory conditions can be self-propagating, as proteases released from PGP-stimulated neutrophils further enhance collagen degradation and peptide production [48]. Under physiological conditions in the lung, PGP is degraded enzymatically by leukotriene A4 hydrolase (LTA4H), preventing its accumulation. While LTA4H is primarily recognized for generating the pro-inflammatory mediator leukotriene B4, it also exhibits aminopeptidase activity, enabling it to cleave PGP [45]. The acetylation of PGP can occur chemically in the lung as a result of reactive aldehydes present in cigarette and wildfire smoke, or generated endogenously during lipid peroxidation [49,50]. Notably, cigarette smoke also selectively abrogates the peptidase activity of LTA4H, likely mediated by reactive aldehydes [51]. While LTA4H readily degrades PGP, acetylation renders the tripeptide resistant to cleavage by this enzyme. Ac-PGP is instead degraded specifically by angiotensin-converting enzyme (ACE). In murine models of intranasal lipopolysaccharide (LPS)-induced acute lung inflammation, ACE expression is upregulated, promoting inflammatory resolution through Ac-PGP degradation. In contrast, in chronic inflammatory conditions, such as COPD, lung tissue from patients exhibits reduced pulmonary ACE levels, which are associated with Ac-PGP accumulation and sustained inflammatory signaling [52]. Together, these findings indicate that the pulmonary microenvironment provides both the proteolytic machinery and chemical conditions necessary for active PGP and Ac-PGP formation, accumulation, and degradation.

### 4.3. Evidence for PGP Activity in the Lungs and on Lung Cells

In the lung, PGP and Ac-PGP exert multiple biological effects primarily through CXCR1/2-dependent mechanisms (reviewed in [46]). For example, PGP recruits and activates neutrophils, contributing to tissue inflammation and damage, as demonstrated by intratracheal administration in mice, which induces neutrophilic inflammation and emphysema-like pathological features consistent with mechanisms implicated in COPD [47]. Ac-PGP increases vascular permeability by phosphorylation of endothelial cadherin in cultured human endothelial cell monolayers, an effect blocked by CXCR2 antagonism [53]. While their pro-inflammatory role in the lungs is prominent, PGP/Ac-PGP can also exert protective effects under specific conditions. For example, intratracheal administration of Ac-PGP reduces pathology, as measured by histological scoring and collagen deposition, in a murine model of bleomycin-induced pulmonary fibrosis, suggesting that this matrikine may modulate fibroproliferative responses [52]. These findings indicate that in the lung, PGP/Ac-PGP function as both mediators of inflammation and regulators of tissue repair. Similarly, in cutaneous wound-healing models in both rats and mice, topical application of Ac-PGP accelerates wound closure and promotes neovascularization. In vitro, Ac-PGP also enhances the migration, proliferation, and tube-forming capacity of human endothelial progenitor cells [54]. Collectively, these observations indicate that both PGP and Ac-PGP are increasingly appreciated not only for their pro-inflammatory properties in peripheral tissues but also for context-dependent protective roles, including modulation of endothelial cell function [34,46].

### 4.4. Evidence for Systemic Activity of PGP

Published studies show that Ac-PGP not only enters the circulation but also exerts systemic biological effects. Ac-PGP is detectable in the serum of healthy humans, with reported concentrations of 6.3 pg/mL in adults and 18.7 pg/mL in newborns [55]. In mice, subcutaneous administration of Ac-PGP exerts systemic effects: in a cecal ligation and puncture-induced polymicrobial sepsis model, it modulates neutrophil activation and cytokine production, reduces apoptosis of thymocytes and splenocytes, and improves survival, with protective effects abolished in CXCR2-deficient animals [56]. Similarly, subcutaneous administration of Ac-PGP induces vascular leak, whereas systemic administration of RTR (arginine-threonine-arginine), which sequesters and inhibits PGP-containing peptides, attenuates LPS-induced pulmonary vascular permeability in vivo [53]. Together, these observations underscore the ability of Ac-PGP to exert biological effects beyond its site of generation and to circulate and influence systemic immune and vascular processes.

### 4.5. Evidence PGP Can Cross the Blood–Brain Barrier

PGP is one of the few matrikines that has been directly demonstrated to cross an intact BBB, as evidenced by increased radioactivity in brain tissue following intravenous, intraperitoneal, or intranasal administration of radiolabeled PGP in rats [57]. In addition, indirect evidence indicates that this matrikine may modulate barrier integrity and further facilitate its CNS access under conditions of BBB disruption. Ac-PGP, in a CXCR2-dependent manner, increases permeability in human endothelial monolayers, which share functional characteristics with vascular barriers, including the BBB [53]. In vivo, PGP produces anxiolytic-like effects in the Vogel conflict test following intraperitoneal administration in rats, indicating engagement of central neural circuits, either through direct CNS access or peripheral-to-brain signaling mechanisms [58]. Because pathological states such as ischemic stroke, neuroinflammation, and systemic inflammation are associated with compromised BBB (reviewed in [25,26]), circulating PGP could plausibly gain CNS access under these conditions and contribute to neuroimmune signaling. Further experimental validation is required to establish this mechanism.

### 4.6. Known Effects of PGP on Brain Cells

There is preliminary evidence that PGP and its acetylated form exert CNS-specific effects, particularly in the regulation of neuronal viability. Martynova et al. [59] initially reported that PGP significantly reduced death of rat pheochromocytoma PC-12 cells treated with H_2_O_2_ at cytotoxic concentrations. PC-12 cells are commonly used as an in vitro model of neurons. A similar neuroprotective effect of PGP is reported in a recent study by Bakaeva et al. [60] who use mechanical scratch-induced injury model in primary rat neuronal cultures to demonstrate that this tripeptide reduces cellular damage measured by two independent cell viability assays. PGP is also shown to inhibit extracellular release of neuron-specific enolase (NSE), a marker of neuronal injury. In addition to its protective effects, this study demonstrates the neuroregenerative potential of PGP, evidenced by a higher number of viable cells within the scratched area of PGP-treated cultures 3–7 days after injury compared with untreated control samples. Application of PGP is also associated with accelerated repopulation of the damaged area by neuronal processes. Additionally, Bakaeva et al. [60] provide insight into the potential molecular mechanisms underlying the neuroprotective and neuroregenerative effects of PGP. Its prophylactic application prevents the injury-induced rise in intracellular Ca^2+^ concentration and the acute loss of mitochondrial membrane potential (ΔΨm) at the time of mechanical damage. Treatment with PGP following injury attenuates disruptions in calcium homeostasis and reduces neuronal cell death. Exposure to PGP is also associated with increased intracellular levels of brain-derived neurotrophic factor (BDNF), suggesting engagement of endogenous neurotrophic support pathways [60].

In contrast to the neuroprotective effects of PGP, its acetylated form is directly neurotoxic to cultured primary rat cortical neurons. Hill and Nemoto [61] report that Ac-PGP activates extracellular signal-regulated kinase 1/2 (ERK1/2) and induces neuronal apoptosis associated with caspase-3 cleavage. Both the selective CXCR2 antagonist SB225002 and a CXCR2-blocking antibody protect neurons from Ac-PGP-induced apoptosis, indicating that the neurotoxic effects of Ac-PGP are mediated, at least in part, by CXCR2-dependent signaling [62]. Because studies directly comparing Ac-PGP with unacetylated PGP are lacking, it remains unclear whether the observed differences in their effects on neuronal viability are attributable to acetylation of the tripeptide, a question that warrants further investigation. Although differences in bioactivity between the two peptides have been reported in peripheral systems (e.g., [44]), no studies to date have examined this issue in the context of their effects on CNS cells. Another largely unexplored area of PGP peptide biology is their potential effects on non-neuronal CNS cells and on more complex neurobiological processes.

It is well established that CXCR2, the primary receptor for both PGP and Ac-PGP, is expressed by all major CNS cell types, including neurons, microglia, astrocytes, and oligodendrocytes. The CXCL1/CXCR2 axis is now recognized not only as a physiological signaling mechanism in the nervous system but also as a contributor to numerous neuropathological processes (reviewed in [62,63,64]). It is therefore plausible that PGP and its acetylated form exert additional, as yet undiscovered effects on both neuronal and non-neuronal CNS cell types. To the best of my knowledge, only a single study has directly investigated the effects of PGP-derived peptides on CNS glial cells. Using the mechanically induced neuronal injury model described above, Bakaeva et al. [60] demonstrate that, in areas adjacent to the scratch, PGP not only exerts neuroprotective effects but also reduces astrocyte hypertrophy and reactivity, as assessed by immunocytochemical staining with anti-glial fibrillary acidic protein (GFAP) antibodies. Furthermore, treatment of the injured neuroglial cultures with PGP leads to a decrease in astroglial scar formation, with astrocyte cell bodies appearing smaller and their processes markedly thinner. Future studies should investigate the effects of both forms of PGP on all glial cell types expressing CXCR1 and CXCR2 across different species to uncover their cell type-specific signaling mechanisms, functional consequences, and potential contributions to CNS homeostasis and pathology.

### 4.7. Possible Contributions of PGP to Neuropathologies

Building on a well-established body of work in peripheral tissues, early evidence suggests that Ac-PGP may also play a pathophysiologically relevant role in CNS disorders by promoting neutrophil recruitment and inflammation, as well as by driving MMP upregulation within the brain parenchyma. Initially, Ac-PGP was identified as a chemoattractant for polymorphonuclear leukocytes (PMNs) released from alkali-degraded rabbit corneas. It also induced chemotaxis and promoted a polarized phenotype in primary human PMNs [65]. Subsequent studies show that injection of synthetic Ac-PGP into normal rabbit corneas mimics the early PMN infiltration observed in alkali-injured eyes, confirming the role of Ac-PGP as an inflammatory mediator [66]. Using CXCR2-deficient mice, later work further establishes that this chemotactic response is dependent on CXCR2, identifying this receptor as a key mediator of Ac-PGP-induced neutrophil recruitment [47]. Similar to its acetylated form, PGP is also shown to induce chemotaxis of human PMNs, an effect that is completely blocked by the combined application of anti-CXCR1 and anti-CXCR2 antibodies [67]. These early studies of the chemotactic properties of PGP in peripheral tissues are directly relevant to its potential contributions to certain CNS pathologies. For example, rapid neutrophil recruitment has been observed in mice after induction of experimental stroke. Neumann et al. [68] demonstrate that in stroke-affected brain regions, neutrophils roll, firmly adhere, and transmigrate at sites of endothelial activation. This ensuing neutrophil invasion is associated with local BBB disruption and the formation of infarcts in the affected brain areas. Notably, in a separate study, Hill and Nemoto [61] report a twofold increase in Ac-PGP levels in the brain tissue of a rat stroke model following middle cerebral artery occlusion (MCAO) compared to sham-operated controls. Since plasma levels of this tripeptide do not differ between the two groups, the authors suggest Ac-PGP is produced locally in the affected brain, likely as a result of MMP activation triggered by the initial injury [61]. Given its direct neurotoxic effects reported in the same study, the elevated levels of Ac-PGP may contribute to further neuronal damage, while its chemotactic properties likely facilitate the recruitment of neutrophils observed not only in animal models of stroke but also in human patients [69].

Neutrophil recruitment during inflammatory responses in both the CNS and peripheral tissues frequently leads to the formation of neutrophil extracellular traps (NETs). In addition to stroke, neutrophil infiltration into affected brain regions has been implicated in the progression of several other neuroinflammatory conditions, including multiple sclerosis, AD, and amyotrophic lateral sclerosis (reviewed in [70]). Of particular relevance to ECM remodeling, activated neutrophils and NETs are enriched in proteolytic enzymes, including neutrophil elastase and MMPs, such as MMP-9. These enzymes can degrade ECM components, thereby facilitating the release of matrikines, including PGP, at sites of inflammation (reviewed in [71,72]). Although such a feed-forward mechanism, whereby NET-associated proteases promote further matrikine generation and sustained neutrophil recruitment, has not yet been directly demonstrated in CNS diseases, the contribution of NETs to the pathophysiology of stroke and other neuroinflammatory conditions has been increasingly recognized [72]. In addition to neutrophils, other CXCR2-expressing CNS cells, including neurons, microglia, and astrocytes, produce MMP-9 and other proteases capable of generating matrikines from ECM proteins. Microglia and astrocytes have also been shown to release the PGP-generating enzyme, prolyl endopeptidase. These processes may be amplified by inflammatory stimuli and under pathological conditions, such as AD (reviewed in [73,74,75,76]). Future studies should investigate whether NET-associated and CNS cell-derived MMPs and other proteases act synergistically to generate PGP and other matrikines in the injured brain, and how this potential feed-forward mechanism contributes to neuroinflammation, neuronal damage, tissue repair, and disease progression in CNS pathologies. Importantly, studies using human cells and in vivo or organotypic tissue models will be critical to validate these mechanisms and assess their relevance to human CNS disease.

### 4.8. Summary: Collagen-Derived PGP Peptides as Candidate Mediators of Lung-to-Brain Signaling

PGP is a collagen-derived matrikine generated in the lung predominantly during inflammatory injury, where it regulates neutrophil recruitment and ECM remodeling via CXCR1/2 signaling. In pulmonary disease, both PGP and Ac-PGP are released into the circulation, providing a mechanistic route by which this lung-derived inflammatory signal may influence the CNS. Under conditions of BBB dysfunction, circulating PGP peptides may access the brain parenchyma and cerebrovascular compartments. PGP may further promote barrier permeability, facilitating its own CNS entry. Notably, the effects of PGP and Ac-PGP on neuronal cells have been examined in only a small number of studies to date, and direct comparative analyses remain lacking. Within the CNS, unacetylated PGP has been associated with neuroprotective and neuroregenerative effects, whereas Ac-PGP promotes CXCR2-dependent neuronal apoptosis and may exacerbate neuroinflammation. However, these apparently divergent actions require confirmation across additional neuronal models, species, and experimental paradigms. Moreover, Ac-PGP-driven neutrophil recruitment, potentially reinforced by NET formation and MMP production by neutrophils as well as CNS-resident cells, suggests a feed-forward lung-to-brain inflammatory loop that amplifies matrix degradation and neural injury. Together, these observations position PGP as a candidate mediator linking pulmonary inflammation to downstream CNS pathology along the lung–brain axis.

## 5. VGVAPG as a Candidate Lung-to-Brain Matrikine

### 5.1. Overview of VGVAPG

Elastin-derived matrikines, known as elastokines or EDPs, share a conserved XGXXPG motif and function as bioactive signaling molecules, exerting diverse effects across multiple organ systems [77,78]. Among these, the hexapeptide VGVAPG is the most extensively studied. It is encoded within exon 24 of human tropoelastin and appears six times in the precursor protein [79]. EDPs, including VGVAPG, are generated by enzymatic cleavage of elastin fibers, primarily by MMPs, neutrophil elastase (also known as leukocyte elastase), and proteinase 3 (Pr3) [34,80,81,82]. In healthy tissues, elastin is highly stable with a half-life of 70–80 years, but during inflammation, aging, or disease, elastin-degrading enzymes can accelerate its breakdown, releasing bioactive fragments such as VGVAPG, which can be found not only as free hexapeptides but also as part of longer elastin fragments or short oligomers containing repeated VGVAPG motifs (reviewed in [83]). VGVAPG, along with other EDPs generated during elastin proteolysis, has been detected in biological fluids, suggesting it is relatively resistant to rapid degradation in vivo. Although the enzymes responsible for its further degradation are not yet identified, VGVAPG appears relatively resistant to proteolysis; for example, it is not hydrolyzed by neutrophil elastase, reflecting the general stability of small elastin fragments in tissues [84]. Once liberated, VGVAPG engages heterotrimeric elastin receptor complexes comprising elastin-binding protein (EBP), neuraminidase-1, and cathepsin A, triggering signaling cascades that engage the aryl hydrocarbon receptor (AhR) and peroxisome proliferator-activated receptor gamma (PPARγ) [85,86,87]. These pathways regulate diverse cellular functions, including immune responses, metabolism, and autophagy [88,89,90]. Increasing evidence suggests that elastokines generated in the lungs act locally, enter the systemic circulation, and influence distal organs, potentially including the CNS.

### 5.2. Evidence of VGVAPG Production in the Lungs

The lungs are rich in elastin and therefore represent a key site for VGVAPG generation during elastin degradation. Although EDPs can be readily detected in the plasma of children and adults [91,92], pathological conditions such as COPD or emphysema cause extensive ECM remodeling, leading to increased elastin degradation and the release of VGVAPG and other elastin fragments into tissues and systemic circulation [32,93,94]. This process is driven by elevated expression and activity of NE, Pr3, and MMPs in the lungs of current smokers and patients with inflammatory lung diseases, including COPD and emphysema, providing a sustained source of these peptides [95,96]. Indeed, significantly higher levels of EDPs and other elastin degradation fragments have been measured by enzyme-linked immunosorbent assays (ELISA) in the sera of COPD or emphysema patients compared with both smokers and non-smoker controls [45,91], while in a separate study, EDP concentrations are shown to be elevated in unconcentrated bronchoalveolar lavage (BAL) fluid from current smokers compared with age-matched non-smokers [97]. These findings suggest that lung injury and chronic inflammation drive EDP production and accumulation, positioning the pulmonary ECM as a major reservoir of elastin-derived bioactive peptides, potentially including VGVAPG (reviewed in [34]).

### 5.3. Evidence VGVAPG Acts in the Lungs and on Lung Cells

VGVAPG exerts multiple biological effects in pulmonary tissues. Its pro-inflammatory activity has been demonstrated by endotracheal administration in mice, which induces an emphysema-like phenotype and increases the proportion of IL-17A- and interferon (IFN)-γ-expressing CD4+ T cells in the lungs and mediastinal lymph nodes [98]. EDPs, including the hexapeptide VGVAPG and κ-elastin (a partially hydrolyzed elastin preparation enriched in EDPs), have been shown to upregulate MMPs. Specifically, MMP-2 and MMP-14 expression is increased in fibrosarcoma cells, whereas MMP-1 is upregulated in human endothelial cells [99,100]. This supports a potential positive feedback loop, in which elastin degradation generates bioactive fragments that promote further MMP expression and elastin turnover and may additionally compromise pulmonary tissue integrity. Moreover, because MMP-2 modulates epithelial and endothelial barrier permeability, elevated levels of this enzyme could facilitate the dissemination of VGVAPG and other EDPs beyond the lung [101]. Pro-inflammatory activity of VGVAPG and other EDPs in the lungs has been attributed to their chemotactic effects on fibroblasts and monocytes, promoting inflammatory cell recruitment and amplifying local tissue responses [34,102,103]. However, in addition to these pro-inflammatory effects, VGVAPG can also exhibit anti-inflammatory activity, for example, by upregulating IL-4 release from CD4+ T cells in COPD patients, but not in control subjects [104]. Furthermore, in mice, this hexapeptide exhibits antithrombotic properties [105] and, in human A549 lung cancer cells, modulates cell cycle and proliferation markers, including Ki-67 and p53 [106], suggesting roles in tissue repair and oncogenic processes in the lung, and highlighting its multifaceted contribution to pulmonary and systemic homeostasis.

### 5.4. Evidence VGVAPG Reaches Circulation and Acts Systemically

Although circulating VGVAPG levels have not been directly measured, several studies have used ELISA or Western blotting to detect EDPs in serum and reported their elevation in patients with COPD, idiopathic pulmonary fibrosis (IPF), and lung cancer [92,107,108,109]. In contrast, serum EDP levels are not different between stroke patients and healthy controls [92], suggesting that elevated circulating EDPs may be more specifically associated with pulmonary pathology. Collectively, these observations raise the possibility that circulating VGVAPG, as a component of the broader EDP pool, may be increased in certain lung pathologies and could potentially interact with distal targets, including within the CNS. Its relative resistance to proteolytic degradation and capacity to act at distant sites are supported by in vivo studies demonstrating that chronic intravenous administration of κ-elastin or VGVAPG in mouse models of atherosclerosis promotes plaque formation [110].

### 5.5. Evidence VGVAPG Can Cross the Blood–Brain Barrier

There is currently no evidence that VGVAPG traverses the intact BBB, but, as noted above, short peptides like this EDP may cross when the BBB is compromised by trauma or gastrointestinal, pulmonary, or neurological pathologies (reviewed in [5,21,25,26]). Notably, VGVAPG has been detected in the CSF of healthy individuals, though it remains unclear whether this peptide originates from the periphery or is generated locally from brain ECM. Nevertheless, the detection of EDPs in CSF at concentrations nearly ten-fold lower than in serum suggests a predominantly peripheral origin with limited but measurable CNS access, supporting the hypothesis that circulating VGVAPG may reach the brain [92]. Furthermore, EDPs have been shown to upregulate MMP-2 in fibrosarcoma and glioblastoma cells [99,111]. If similar effects occur in endothelial cells, and given that MMP-2 can increase endothelial permeability and compromise the BBB [101], VGVAPG may access the CNS not only under conditions of pre-existing barrier impairment but also by actively promoting its disruption.

### 5.6. Known Effects of VGVAPG on Brain Cells

Although relevant evidence remains limited, it appears that once within the CNS, VGVAPG may influence neuronal and glial cell function. Multifaceted effects on several neuronal cell types have been reported. In human SH-SY5Y neuronal cells, VGVAPG upregulates autophagy-related genes *MAP1LC3B* (encoding microtubule-associated protein 1 light chain 3 beta) and *SQSTM1* (encoding sequestosome 1, p62). Further experiments using the agonist rosiglitazone and the antagonist GW9662 confirm that this peptide acts by engaging the PPARγ pathway, modulating mechanistic target of rapamycin (mTOR) and ultimately autophagy [90]. While autophagy can be neuroprotective in acute settings, excessive activation is associated with neuronal damage. Consistent with this, VGVAPG treatment of SH-SY5Y cells reduces α-tubulin acetylation, which correlates with neurite shortening, suggesting microtubule destabilization and subsequent neuronal injury. In addition, VGVAPG-induced downregulation of the phosphoinositide 3-kinase-protein kinase B (PI3K-AKT) pathway via phosphatase and tensin homolog (PTEN) activation leads to glycogen synthase kinase 3 β (GSK3β) disinhibition and Tau hyperphosphorylation in differentiated SH-SY5Y cells, providing a molecular mechanism that may contribute to neuronal damage and potentially increase the risk or accelerate the progression of neurodegenerative diseases [112,113]. Similarly, Ma et al. [114] report neurotoxic effects of VGVAPG in murine models of neurodegeneration. Using murine HT22 neural cells and primary hippocampal neurons, they show that VGVAPG reduces neuronal viability and alters cell morphology. Immunostaining for postsynaptic density protein 95 (PSD95) in primary neuronal cultures reveals a significant reduction in the number of PSD95 puncta, indicating impaired synaptic integrity. VGVAPG also induces upregulation of galectin-3 (Gal-3) in HT22 neuronal cells, a protein associated with neuronal injury that promotes recognition and removal of damaged neurons by microglia.

Ma et al. [114] also demonstrate that VGVAPG directly modulates microglial function. Using murine BV-2 microglia-like cells, they show that VGVAPG induces a reactive phenotype characterized by a more ameboid morphology, increased phagocytic activity, and upregulation of mRNA for several key markers, including *Tgfb1* (transforming growth factor β1), *Il10* (IL-10), *Cd206/Mrc1* (mannose receptor C type 1), *Il1b* (IL-1β), and CD86. Importantly, intracerebroventricular injection of VGVAPG in 6-month-old mice leads to increased microglial activation in the hippocampal CA1 region, as indicated by higher density of ionized calcium-binding adapter molecule 1 (Iba1)-positive cells exhibiting enlarged cell bodies and simplified branching. This is accompanied by substantial dendritic spine loss, reduced synaptic integrity, and increased colocalization of PSD95 within Iba1-positive microglia, suggesting that VGVAPG promotes microglia-mediated synaptic pruning and neuroinflammatory changes in the hippocampus. Collectively, these findings indicate VGVAPG-induced synaptic and neuronal damage in vivo.

Astrocytes represent an additional cellular target through which VGVAPG may contribute to neurodegenerative processes, although the reported effects on this cell type appear context-dependent. In murine primary astrocytes, VGVAPG reduces endothelial nitric oxide (NO) synthase (eNOS), inducible NO synthase (iNOS), and neuronal NO synthase (nNOS) protein expression. Consistent with these effects, VGVAPG treatment decreases NO production while increasing reactive oxygen species (ROS) generation, indicating a shift toward an oxidative stress state in astrocytes [115]. The effects of VGVAPG on astrocytes do not appear to be pro-inflammatory since it increases caspase-1 activity in astrocytes but simultaneously decreases the release of IL-1β into the cell-culture medium. ELISA reveals that in vitro treatment with VGVAPG increases the protein expression of superoxide dismutase 1 (SOD1) in astrocytes, whereas it decreases the expression of IL-1β receptor 1 (IL-1βR1), catalase (CAT), and nuclear factor (NF)-κB [116]. However, VGVAPG engages AhR-dependent signaling and differentially modulates the expression of genes involved in metabolism, inflammation, and proteostasis, including *Sirt3* (sirtuin 3), *Pparg* (PPARγ), *Nfkb1* (NF-κB subunit 1), *Ide* (insulin-degrading enzyme), and *Mme* (membrane metalloendopeptidase, neprilysin) [86]. Interestingly, in vitro exposure of primary murine astrocytes to VGVAPG leads to downregulated mRNA expression of *MMP-2* and *MMP-9*, but upregulated expression of mRNA for tissue inhibitors of metalloproteinase (*TIMP*)*-1* and *TIMP-2* [117]. This suggests that, as in the lungs, VGVAPG may modulate ECM remodeling in the CNS, potentially influencing elastin turnover and generation of EDPs. Taken together, these findings highlight the multifaceted effects of VGVAPG on neurons, microglia, and astrocytes, but studies in humans and across other CNS cell types, particularly oligodendrocytes, are largely lacking, underscoring the need for further research to define its contribution to brain homeostasis and neuropathology.

### 5.7. Possible Contributions of VGVAPG to Neuropathologies

EDPs have been implicated in diverse peripheral pathologies including atherosclerosis, cancer, nonalcoholic fatty liver disease, lung emphysema, and COPD (reviewed in [118]). Notably, a linear regression analysis indicates a significant positive correlation between concentrations of matrikines derived from several different ECM proteins, including elastin, and age in both humans and mice, suggesting that the accumulation of these peptides may reflect age-related ECM remodeling and tissue senescence [103]. In contrast, the contribution of EDPs to neuropathologies is only beginning to emerge, and direct studies examining VGVAPG specifically in human neurological disease remain scarce (reviewed in [36,119]). Nonetheless, VGVAPG and related elastin-derived sequences may contribute to neuropathology through pro-inflammatory, synaptotoxic, and barrier-modulating mechanisms described above. Clinical support for central involvement comes from Nicoloff et al. [92], who use ELISA and Western blotting to demonstrate a more than 20-fold increase in EDP concentration in CSF from patients with acute cerebral infarction compared with healthy controls. The authors propose that elevated CSF levels may result from leakage from affected cerebral vessels or passive diffusion from serum. However, the latter explanation appears less plausible, as the marked increase in CSF-derived peptides is not accompanied by any change in their serum concentrations. The same research group reports a similar increase in CSF levels of EDPs when three different subgroups of stroke patients are considered, including lacunar stroke, non-lacunar ischemic stroke, and recurrent stroke [120]. Relevant to the potential role of elastin degradation and EDPs in AD are the in vitro and in vivo findings reported by Ma et al. [121], who employ elastin-like polypeptides (ELPs) composed of repetitive canonical elastin sequences. These ELPs consist of low-complexity pentapeptide repeats of the motif (VPGVG)_n_, and the study utilizes constructs of varying backbone lengths, specifically ELP30, ELP48, and ELP90. In vitro treatment of a Chinese hamster ovary cell line stably expressing mutant human amyloid precursor protein (APP) with ELPs results in significant overproduction of amyloid β peptides. Furthermore, two months after intracerebroventricular injection of ELPs in mice, there is an increased immunostaining signal for phosphorylated Tau protein at serine 396, as well as elevated levels of amyloid β peptides—both pathological features associated with AD [121]. The marked elevation of EDPs in CSF, in the absence of corresponding increases in serum, further raises the possibility that elastin degradation occurs locally at the level of the cerebral vasculature or is facilitated by BBB disruption [92,120]. In this context, a feed-forward mechanism is plausible in which vascular injury and ECM degradation promote the generation and release of EDPs, while EDP-induced upregulation of MMPs [99,100] and the established role of MMPs in modulating endothelial barrier permeability [101] further compromise BBB integrity and contribute to progressive barrier dysfunction. Such a cycle could amplify both matrikine accumulation within the CNS and the progression of neurovascular and neurodegenerative pathology, although direct experimental validation of this mechanism remains limited. Collectively, these findings highlight a compelling, yet insufficiently explored, link between elastin degradation products and CNS pathology, underscoring the need for systematic mechanistic and translational studies to define the role of VGVAPG and other EDPs in human cerebrovascular and neurodegenerative diseases, as well as in other neuropathologies.

### 5.8. Summary: Elastin-Derived VGVAPG as Candidate Mediator of Lung-to-Brain Signaling

EDPs, including the hexapeptide VGVAPG, are bioactive matrikines generated through enzymatic degradation of elastin, particularly during inflammation, aging, and tissue remodeling. Produced abundantly in elastin-rich organs such as the lungs, VGVAPG is released into the local extracellular milieu and pulmonary interstitium and can subsequently enter the circulation in conditions such as COPD and emphysema, where elevated MMP and neutrophil elastase activity accelerates ECM breakdown. In pulmonary tissues, VGVAPG exerts pleiotropic effects, including pro-inflammatory signaling, modulation of MMP expression, and regulation of immune cell activity and recruitment, while also displaying context-dependent anti-inflammatory and antithrombotic properties. Circulating EDPs have been detected in serum and CSF, suggesting that lung-derived peptides may act systemically and potentially access the CNS, particularly under conditions of dysfunctional BBB, where they may both exploit and further exacerbate barrier impairment. Experimental studies demonstrate that VGVAPG influences multiple CNS cell types. In neurons, it activates PPARγ-dependent autophagy pathways, promotes Tau hyperphosphorylation, reduces synaptic markers, and induces neurite shortening. In microglia, it triggers a reactive phenotype and enhances phagocytic activity, contributing to synaptic pruning in vivo. Astrocytes exhibit altered redox balance and ECM remodeling responses. Clinical observations of elevated EDP levels in stroke patients and AD-like pathology induced by elastin-like polypeptides in animal models further support their potential role in neurodegeneration. Collectively, these findings implicate VGVAPG as a possible mechanistic link between pulmonary elastin degradation and CNS pathology, warranting further translational investigation. To provide a concise overview of the cellular targets, receptors, downstream pathways, and functional effects of the primary lung-to-brain matrikines reviewed, relevant information for PGP, Ac-PGP, and VGVAPG is summarized in Table 1.

While ECM-derived matrikines such as PGP and VGVAPG provide a mechanistically compelling link between lung ECM remodeling and CNS effects, several limitations should be acknowledged. Most available data derive from in vitro or animal studies, and evidence supporting these matrikines as human lung-to-brain signaling molecules remains limited. The biological effects of these peptides are context-dependent, varying with disease state, peptide concentration, and timing, and some matrikines exhibit pleiotropic or even opposing effects, which may complicate their translation into therapeutic strategies. Additionally, the precise mechanisms governing systemic distribution, including the potential role of extracellular vesicles, remain to be fully defined. Finally, as noted above, the potential role of PGP and VGVAPG in feedback brain-to-lung signaling under pathological conditions is largely unexplored and will require further study.

## 6. Other Candidate Matrikines Linking Lung and Brain

Beyond elastin-derived VGVAPG and collagen-derived PGP species, several additional matrikines exhibit properties consistent with potential lung-to-brain signaling molecules. They include GHK, which is released from matricellular secreted protein acidic and rich in cysteine (SPARC) and type I collagen; laminin-derived IKVAV and YIGSR; and endorepellin, corresponding to domain V of ECM protein perlecan (reviewed in [122,123,124]). All of these matrikines have been shown to modulate at least one CNS cell type, either in vitro or in vivo. For example, GHK enhances the survival of primary murine astrocytes exposed to hemin-induced toxicity via activation of miR-146a-3p and the PI3K-AKT signaling pathway [125] and similarly protects human SH-SY5Y neuroblastoma cells from hemin-induced apoptosis by reducing p38 mitogen-activated protein kinase phosphorylation and downstream miR-339-5p expression [126]. IKVAV suppresses astrocytic differentiation in cultured neural stem cells, potentially impacting brain tissue homeostasis [127], whereas YIGSR mitigates 1-methyl-4-phenylpyridinium (MPP^+^)-induced microglial activation and dopaminergic neurotoxicity in primary murine neuron-glia cultures [128]. Endorepellin enhances neuronal differentiation of murine neural progenitor cells and promotes neurite extension in vitro, consistent with neuroregenerative activity [129].

Experimental evidence demonstrates that two of the above matrikines, GHK and endorepellin, can access the CNS from the circulation, particularly under pathological conditions. Following intravenous injection of radiolabeled GHK in mice, the peptide distributes to multiple tissues, with brain concentrations exceeded only by kidney uptake, indicating passage from the periphery into the CNS [130]. Consistent with central bioavailability, intranasal administration of GHK-Cu to 5xFAD transgenic mice, a model of AD pathology, improves cognitive performance, reduces amyloid plaque burden, and decreases neuroinflammatory markers, including CCL2, in the frontal cortex and hippocampus [131]. Similarly, endorepellin has been documented to reach and exert functional effects within the injured CNS. In rat models of ischemic stroke, systemic administration of recombinant human endorepellin 24 h post-insult limits lesion expansion and restores motor performance to pre-stroke levels for at least 15 days [132]. Complementing these findings, intraperitoneal delivery of endorepellin in a mouse MCAO model increases neural progenitor proliferation in the subventricular zone and dentate gyrus and elevates the number of newly generated neurons in peri-infarct cortex, consistent with enhanced post-ischemic neurogenesis and functional recovery [129]. Together, these findings indicate that peripheral GHK and endorepellin can reach the CNS, engage neural and glial targets, and promote functional recovery and neuroregenerative processes, highlighting their potential as lung-to-brain signaling candidates; however, direct evidence that GHK, IKVAV, YIGSR, or endorepellin are produced or released from the lung in amounts sufficient to enter the circulation and support such signaling is currently lacking. Further research is also required to determine whether IKVAV, YIGSR, and other matrikines can similarly access the CNS and modulate cell-specific functions under physiological or pathological conditions.

## 7. Conclusions and Future Directions

The evolving concept of the lung–brain axis underscores a complex network of bidirectional signaling in which the ECM plays a significant but underexplored role. This manuscript establishes that the lung, with its high elastin and collagen content and susceptibility to intense proteolytic activity, serves as a significant reservoir for matrikines. Evidence strongly supports collagen-derived PGP and elastin-derived VGVAPG as primary candidate mediators of lung-to-brain signaling. These peptides are generated during pulmonary inflammation, enter systemic circulation, and can traverse a compromised BBB to influence CNS homeostasis. Once within the brain parenchyma, these matrikines modulate the functional states of neurons, microglia, and astrocytes, contributing to both neuroprotective and neurotoxic outcomes depending on the specific peptide species and pathological context. Beyond these primary candidates, other matrikines, including GHK and endorepellin, further expand the repertoire of potential lung-derived signaling molecules. Ultimately, these bioactive fragments may represent a critical mechanistic link between chronic respiratory pathologies, such as COPD and emphysema, and the development or progression of neurological disorders, including AD and stroke. The translational potential of matrikine-mediated lung-to-brain signaling may be explored through modulation of CXCR2-dependent pathways. For example, selective CXCR1/2 antagonists such as Reparixin have demonstrated efficacy in reducing neutrophil-driven inflammation in preclinical models and could potentially mitigate Ac-PGP-driven neurotoxicity [47,61]. These approaches highlight the feasibility of targeting specific chemokine-receptor axes to interrupt lung-derived inflammatory signals impacting the CNS. Future research should prioritize definitive in vivo tracing of matrikines, characterization of their cell type-specific effects within the CNS, and human clinical validation to fully delineate these pathways and their therapeutic potential, including in both chronic and acute inflammatory conditions. Although direct evidence linking matrikines to CNS effects in COVID-19 is currently lacking, severe acute respiratory syndrome coronavirus 2 (SARS-CoV-2) infection is accompanied by extensive ECM remodeling and upregulation of MMPs, key proteases responsible for matrikine generation (reviewed in [133]). SARS-CoV-2 infection also frequently leads to neurological manifestations ranging from headache and anosmia to encephalopathy and cognitive dysfunction, reflecting the impact of systemic inflammation and peripheral organ injury on CNS function (reviewed in [134]). This raises the speculative but intriguing possibility that matrikine-mediated signaling may contribute to CNS symptoms observed in COVID-19 and that targeting such pathways could ultimately mitigate virus-associated neuroimmune dysregulation. Investigation of this avenue could therefore provide insights relevant not only to chronic respiratory diseases but also to acute virus-associated neuropathologies.

## Figures and Tables

**Table 1:** Summary of best-supported candidate matrikines for lung-to-brain communication (PGP, Ac-PGP, and VGVAPG): cellular targets, signaling pathways, functional effects, and CNS access.

Matrikine (Protein of Origin)	Cellular Targets	Receptor/ Signaling Pathway	Functional Effects in the CNS	Evidence of CNS Access	Key References
PGP/ Ac-PGP ^1^ (Collagen)	Neutrophils, neurons, microglia, astrocytes	CXCR1, CXCR2/ERK1/2, intracellular Ca^2+^ regulation, apoptotic signaling	Modulation of astrocyte reactivity, potential neuroprotective effects (PGP); neutrophil recruitment, MMP upregulation, neuroinflammation, neuronal apoptosis (Ac-PGP)	Yes (PGP crosses BBB; Ac-PGP increases permeability; elevated Ac-PGP in stroke models)	[47,60,61,65,66,67,68]
VGVAPG (Elastin)	Neurons, microglia, astrocytes	Elastin receptor complex (EBP, cathepsin A, neuraminidase-1)/AhR, PPARγ, autophagy modulation	Neuronal damage, neurite shortening, synaptic marker reduction; increased microglial reactivity and phagocytic activity; altered astrocyte redox, metabolic, and inflammatory states, and ECM remodeling	Indirect (elevated CSF relative to serum levels in humans; possibly BBB disruption-dependent)	[79,86,90,92,99,112,113,114,115,116,117]

^1^ Ac-PGP, N-acetylated proline-glycine-proline; AhR, aryl hydrocarbon receptor; BBB, blood–brain barrier; CNS, central nervous system; CSF, cerebrospinal fluid; CXCR, C-X-C motif chemokine receptor; EBP, elastin-binding protein; ECM, extracellular matrix; ERK, extracellular signal-regulated kinase; MMP, matrix metalloproteinase; PGP, proline-glycine-proline; PPARγ, peroxisome proliferator-activated receptor gamma; VGVAPG, valine-glycine-valine-alanine-proline-glycine.

## Data Availability

No new data were created or analyzed in this study. Data sharing is not applicable.
